# Reliability of Auditory-Perceptual Analysis in the Study of Speech Function in Patients with Unilateral Cleft and Palate

**DOI:** 10.3390/jcm15020588

**Published:** 2026-01-12

**Authors:** Alexandra Bloeck, Nora Ann Doyle, Sylva Bartel, Michael Krimmel

**Affiliations:** 1 Practice for Speech and Language Therapy, 55590 Meisenheim, Germany; research@logopaedie-meisenheim.de; 2 Department of Oral and Maxillofacial Surgery, University Hospital Tübingen, 72076 Tuebingen, Germany; nora-ann.doyle@student.uni-tuebingen.de; 3 Department of Phoniatrics and Pediatric Audiology, ENT Clinic, University Hospital, 06120 Halle (Saale), Germany; sylva.bartel@uk-halle.de

**Keywords:** cleft lip and palate, auditory-perceptive analysis, speech reliability

## Abstract

**Background/Objectives**: Multidisciplinary outcome studies are carried out to evaluate long-term treatment in patients with cleft lip and palate. Speech function as one of the key outcomes of the treatment is examined by means of an auditory-perceptual analysis. For scientific and global studies it is essential to reduce the risk of bias as much as possible. The aim of the present study was the examination of auditory-perceptive analyses on the basis of an outcome study. Reliability was evaluated. **Methods**: Twenty patients were examined to evaluate their speech function. The speech sample was obtained via the online tool Zoom™. The speech sample consisted of single words (picture supported), a version of the German “Great Ormond Street Speech Assessment” (GOS.SP.ASS) sentences and spontaneous speech. The analysis was carried out by three experienced examiners, all using the German version of the Universal Reporting Parameters at two different times. The intrarater and interrater reliability were calculated. **Results**: Twenty participants with unilateral cleft and palate and a minimum age of 18 years (ø 20.1) were enrolled in the analysis of the speech function. None of the participants had undergone a secondary operation due to velopharyngeal incompetence. The examination happened at a point in time before an osteotomy might be needed. The multidisciplinary treatment of the 20 participants regarding their speech function was successful. There were only marginal abnormalities. The listeners showed a very good intrarater and moderate interrater reliability (ICC/Fleiss’ kappa). An overall percentual agreement of 88.3% was achieved. **Conclusions**: These positive results cannot be compared with outcome studies on a national or international level, since the construction of the speech sample as well as the structure and the implementation of the auditing process reveal considerable deficiencies in methodological rigor. The small number of examiners and patients as well as the patients’ minor residual impairments influence the significance of the statistical calculation by kappa and ICC. The auditory-perceptual analysis should be validated for German-speaking countries.

## 1. Introduction

Language and speech are vital for social integration. Orofacial impairments caused by cleft lip and palate (CLP) can compromise intelligibility and social acceptance, significantly limiting patients’ societal activity and participation, and thus, quality of life. In many cases the impaired orofacial structures can be sufficiently managed by surgical or orthodontic treatment so that speech quality improves significantly. However, there will still remain patients in need of speech and language therapy [1], which should be applied by specified speech and language therapists (SLTs) [2]. SLTs are part of the interdisciplinary care team for cleft treatment [3]. Although there are several cleft centers in Germany, standards are still lacking when it comes to the structure and quality in interdisciplinary work. In Germany major cleft centers are typically located at university hospitals that comprise an oral maxillofacial surgery and a department of orthodontics. The absence of interdisciplinary interfaces leads to reduced impact of SLTs in German cleft research. In addition, the lack of a general academized education of SLTs in Germany hinders professionals in the field from conducting research. Both factors led to a severe gap in the German research of speech function in patients with CLP.

In addition to genetic and environmental factors, treatment schemes can have a severe impact on speech outcome of the patients [4]. In interdisciplinary work it seems obvious that all disciplines apply evidence-based best practice treatment to supply the best outcome for the patient. The investigation of van Roey et al., focusing on unilateral and bilateral CLP, demonstrated, that surgical treatment protocols vary considerably across Europe [5]. All protocols commonly start with lip closure, then the closure of the soft palate follows early, the hard palate though is undertaken at different times. Most differences are seen in the time of closure of the alveolus. The study could not indicate the preferred surgical techniques due to limited coverage of this aspect in previous studies. The interface with SLT is the quality of surgical closure or the structural characteristics of the remaining tissue, both of which can contribute to resonance disorders in speech function. The orthodontic treatment includes presurgical treatment (e.g., palatal feeding plate, nasoalveolar molding (NAM)) to allow proper alignment of the alveolus before and after surgery. Orthodontics monitor the craniofacial growth and dental development; they prepare the dental arch for surgical intervention and correct malocclusions [3]. The orthognathic structure, the way of occlusion and the position of the tongue can influence the articulation of speech function [6]. To date, to our best knowledge speech therapy interventions have not been systematically evaluated to determine the most appropriate treatment protocol for CLP. Instead, a range of therapeutic approaches is available, which are applied as flexible tools and tailored to the patients individual needs. For patients with deficits in speech function, intelligibility and acceptability speech therapy should use the motor phonetic or the linguistic phonological approach according to the present deficiency [3]. The success of the CLP treatment is altogether interdependent between the disciplines.

Internationally, researchers have examined cleft-type characteristics (CTCs) in speech for at least three decades. With the Great Ormond Street Speech Assessment (GOS.SP.ASS), Sell intended to make speech quality quantifiable in patients with CLP [7]. The standardized auditory-perceptive analysis had been established. Relevant literature formed the basis for making specific variables visible in definitions and distinctions, in certain diacritics of the phonetic alphabet, and in defined scales for generating research data. By means of the GOS.SP.ASS, it is possible to distinguish developmental or other speech impairments from CTCs. At the annual conference of the American Cleft Palate-Craniofacialial Association in 2002, the development of universal and globally applicable test parameters for auditory-perceptive analysis in patients with CLP was initiated, mainly based on the research work of Kuehn and Moller (2000) [8]. Hypernasality, hyponasality, nasal emission/nasal turbulence and articulation errors—filtered out as globally measuring parameters in CLP speech—were merged and defined into the “Universal Parameters for Reporting Speech” (URP) [2] and represent acoustic phenomes as CTCs which appear independently of a patient’s mother tongue in seemingly all languages. They are listed in Table 1. 

The introduction of national data pools for research and quality measurement in cleft management (CRANE und SCANDCLEFT) led to the discussion for improvement in quality criteria of auditory-perceptive analysis. In 2004 Lohmander and Olsen argued in a review, which included 76 studies, that the great heterogeneity of variables [9] had been additionally confounded by incomprehensible research designs and unclear implementations. Many authors then showed that methodical rigor and continuous training can improve reliability [9,10,11].

The Cleft Audit Protocol for Speech–Augmented (CAPS-A) [12] was developed based on the revised GOS.SP.ASS [13] and on the CAPS, which had been used as an internal clinical audit tool in the UK in the late 1990s. Whereas the more detailed GOS.SP.ASS still serves as a solid foundation for therapy management, the CAPS-A acts as a basis for data collection for research projects. In the CAPS-A protocol the speech samples, the implementation and data analysis became standardized. Good reliability was demonstrated [12]. A CAPS-A training program was developed for further optimization [14]. The CAPS-A was translated, standardized and validated in two further languages [15,16]. In Scandinavian countries the SCANDCLEFT project introduced speech samples with single words which had been developed under strict linguistic rules and showed good reliability. These can be found in word lists in eight different languages [17]. With the development and validation of the velopharyngeal sum score [18], Lohmander et al. successfully implemented CTCs in connection with velopharyngeal insufficiency. In 2020 Pereira et al. connected the Scandinavian sum score with the CAPS-A to create the CAPS-A VPC SUM to specify the indication of a velopharyngeal plastic [19].

The informal German GOS.SP.ASS [20] was modified and uploaded with a manual by Neumann on the digital platform Yumpu [21], which is not well known under SLTs who specialize in CLP. Development criteria for sentences or words are not documented. No standardization exists for speech sampling, implementation, or analysis. Today, several versions exist of GOS.SP.ASS sentences or single-word lists in different German-speaking countries and even within different cleft centers. Self-made speech samples are in use [22].

In her publication of the German version of the URP (URP-D) [23], which was used for this study, Neumann did not consistently follow the originally established development conditions published by Henningsson et al. in 2008 [2]. The adaptions of the aforementioned assessments (CLIPSI or CAPS-A, CAPS-A VPC SUM) for improvement in reliability are not implemented in Germany. Consequently, there is a great heterogeneity in definitions of cleft parameters, assessment sheets, speech samples and administration protocols as well as data analysis. A good interrater reliability for national studies can thus hardly be expected, not to mention compatibility with global studies.

The research question for this study was as follows:

How robust is the intra- and interrater reliability of the auditory-perceptual analysis using the German GOS.SP.ASS sentences and the German URP-protocol in the assessment of patients of the German population with unilateral cleft lip and palate in the present outcome study?

## 2. Materials and Methods

For the present study, the patient charts at the Department of Oral and Maxillofacial Surgery at the University Hospital Tübingen were systematically searched to identify all patients with a diagnosed unilateral complete CLP. Inclusion criteria were as follows: patients had to be born between 1 January 2000 and 31 December 2005 and be at least 18 years old at the time of evaluation. Surgery had to be exactly defined and was performed only by the cleft team at the Tübingen hospital. The soft palate of all patients was operated in the technique of Kriens.

Patients had to have attended follow up examinations regularly. None of the patients had undergone midface osteotomy prior to this point or had had speech-improving surgery. Patients were excluded if there were syndromes or further comorbidities, missing data, or prior operations or care at a different clinic. A total of 42 patients were identified from the data pool. After applying exclusion criteria, 20 persons were left to become part of the study, 11 of whom were male (55%) and 9 female (45%). The average age was 20.1 years; the age range was 18.5–23.0 years. Sixteen (80%) of the participants had a cleft on the left side, four patients (20%) on the right side.

The recruitment interviews followed a consistent procedure, beginning with an initial telephone conversation, followed by information about the study’s objective, procedure and estimated duration of participation, the voluntary nature of the study, pseudonymization of data and right to withdraw at any time. The study was accepted by the ethics commission of the medical faculty of the Eberhard-Karls-Universität Tübingen on 19 December 2023 under the project number 745/2023BO2.

Patients had to give informed consent once more shortly before data collection for the auditory-perceptive analysis. The examination took place on Zoom™ (version 5.17.0, San Jose, CA, USA) between 14 January 2024 and 20 April 2024 and was carried out by a PhD student of the medical faculty of the University Tübingen. The researcher always performed the examination in the same room at the same PC. The participants took part in their homes from their PC or laptop. The examination conditions on the part of the participants were therefore not standardized and corresponded to their respective setups. The participants needed to consent again shortly before recording. Five of the patients consented to the audio only; all the others consented to audio and video recording. All speech samples obtained were analyzed.

The speech sample consisted of a non-standardized German version of the GOS.SP.ASS with single words, sentences and a guided interview with questions to document connected speech (see Table S1). The analysis of the Zoom™ audio and video recordings was carried out by two experienced examiners from a different university hospital and by an examiner working in private practice who has completed the CAPS-A training course. The analysis was conducted twice at an interval of 4–6 weeks. The listeners gathered data via an auditory-perceptive analysis. When participants consented to video recording, the listening process was augmented by visual observation. The observation served as a supplement to the auditory analysis, although it was not analyzed separately. The auditory-perceptive analysis was based on the conditions of the LKGSF-Komplex [23], which is linked to Henningsson et al. [2]. The data was documented with the data collection sheet 2 of the URP_D (see Table S2). The medical history or other relevant medical data were not known to the listeners. Raw data was entered into an Excel™ table. Analysis was merely descriptive due to the low numbers of both participants and listeners.

The URP data collection sheet includes two different scale levels. The Intraclass Coefficient (ICC) with Two-Random Effect Model (absolute agreement, single rater/measurement) (ICC 2.1) was used to calculate the interrater reliability of the parameters with ordinal scales [24]. Fleiss‘ kappa [25] was calculated for individual measurement of the parameters with nominal scales (hyponasality and voice disorder).

Intrarater reliability was assessed using an ICC based on a Two-Way Mixed-Effects Model (single measurements and consistency), corresponding to ICC (3.1) [24]. The nominal scales (hyponasality and voice disorder), rated by three independent raters, were assessed using weighted kappa [26].

For all parameters (including consonant production) percentual agreement was calculated by individual measurement and by the average of all values. Everything was calculated using Excel™ (version 16.104 (25121423), Washington, DC, USA) except for intrarater reliability, which was conducted using Copilot (Washington, DC, USA) [27]. The clinical relevance in connection with Fleiss’ kappa, weighted kappa, ICC or percentual agreement is listed in Table 2.

## 3. Results

The results for hypernasality, nasal emission/nasal turbulence, intelligibility and acceptance are listed in Table 3; the results for hyponasality and voice are in Table 4; and the results for consonant production errors in Table 5. The raw values show the actual number of persons (n = 20) who could be identified in the different categories.

### 3.1. Hypernasality

Hypernasality was evaluated across the domains of single words, sentences, and spontaneous speech, using the categories *within normal limits, mild, moderate, and severe*. All raters classified 19 participants as *within normal limits* in single-word production, with 1 participant rated as exhibiting *mild* hypernasality (ICC = 0.31, 100%).

For sentence-level speech, 16 participants were rated as *within normal limits*, 3 to 4 participants as *mildly* hypernasal, and 1 participant as *moderately* hypernasal (ICC = 0.54, 95%). In spontaneous speech, 16 participants were rated as *within normal limits*. Between three and four/five participants were classified as *mildly* hypernasal, while one participant was rated as *moderately* hypernasal by the raters R1 and R2 (ICC = 0.54; 95%).

### 3.2. Nasal Emission/Nasal Turbulence (NE/NT)

In the URP nasal emission and nasal turbulence are merged into one single variable. NE/NT was evaluated in the domains of single words, sentences and spontaneous speech, using the categories *none/within normal limits, present (intermittent or variable) or present (frequent or pervasive)*. In single words, raters showed deviations between R1/R2 and R3. R1/R2 classified 9 and R3 13 participants as *within normal limits*. R1/R2 rated NE/NT in 11 and R3 in 7 persons as *intermittently present* (ICC = 0.36; 80%). At the sentence level, R1/R2 rated 8 and R3 12/11 participants as *none/within normal limits*. R1/R2 rated 11 and R3 7/8 persons with *present* (*intermittent or variable*) (ICC 0.53; 85%). All raters analyzed one person with *present* (*frequent or pervasive*) (ICC 0.53; 85%). The evaluation of spontaneous speech showed a lower percentual agreement (ICC 0.50; 65%). Deviations occurred between *none/within normal limits* and *present* (*intermittent or variable*).

### 3.3. Intelligibility

Intelligibility was assessed on the basis of spontaneous speech with the categories *within normal limits, mild, moderate and severe*. All raters showed total agreement: 17 participants were rated as *within normal limits*, 3 with mild impaired intelligibility. None of the participants showed moderate or severe deficits (ICC 0.52; 100%).

### 3.4. Acceptability

The acceptability of the speech was based on the whole speech sample and was rated in the same categories as intelligibility. Deviations occurred between R1/R2 and R3. R1/R2 rated 17 and R3 14 participants as within normal limits. R1/R2 rated three persons as mildly impaired and R3 five. R1/R2 rated none and R3 one person as moderately impaired. None of the participants were rated with severely impaired acceptability (ICC 0.20; 85%)

The mean percentual agreement across all parameters (hypernasality, nasal emission/nasal turbulence, intelligibility and acceptability) was 88.13%.

### 3.5. Hyponasality

Hyponasality was evaluated across the domains of sentences and spontaneous speech using the categories *none/within normal limits* and *present*. Ratings between the three listeners showed inconsistencies. In the category sentences R1 rated 17, R2 16 and R3 10 participants as within *none/normal limits*. Hyponasality was rated as present by R1 in 3, R2 in 4 and R3 in 10 participants (k = 0.37; 70%). Similar divergences were found in the evaluation of spontaneous speech (k = 0.77; 80%).

### 3.6. Voice Disorder

Voice disorder was calculated on the basis of the entire speech sample in the categories *none/within normal limits* and *present.* Deviations occurred between R1/R2 and R3. R3 identified three/four persons, R1/R2 both rated one with a present voice disorder (k = 0.83; 90%).

The mean percentual agreement across all parameters (hyponasality and voice disorder) was 80%.

### 3.7. Consonant Production Errors

Consonant production errors were evaluated in the domains of single words, sentences and spontaneous speech in the categories *none/within normal limits* and *present*. Overall, the speech samples of the 20 participants exhibited only residual symptoms, primarily in the areas of *weak articulation* and *other oral misarticulations*. Here, the greatest discrepancies between raters R1/R2 and R3 were observed. For all other parameters, nearly full agreement was achieved (see Table 5).

The mean percentual agreement in consonant production errors was 89.03%.

### 3.8. Interrater Reliability

The interrater reliability for individual measurements across the respective parameters, as measured by ICC or Cohen’s kappa, showed overall moderate to low values. At first glance, these results appear inconsistent with both the raw data and the percentage of agreement. For example, Table 3 shows 100% agreement for hypernasality, yet the ICC indicates only a low level of reliability. Calculating all parameters collectively, the percentage of agreement was 88.9%, which corresponds to a good level of agreement (see Table 2).

### 3.9. Intrarater Reliability

In the evaluation of individual measurements, R1 and R2 each achieved an ICC of 1.000, while R3 obtained an ICC of 0.995. These values were calculated for single measurements based on ordinal scales. For nominal scales R1 achieved a weighted kappa of 1.000, R2 0.818 and R3 0.76. This indicates altogether a very good agreement (Table 6).

## 4. Discussion

The research question of the present study was as follows: How robust is the intra- and interrater reliability of the auditory-perceptual analysis using the German GOS.SP.ASS sentences and the German URP-protocol in the assessment of patients of the German population with unilateral cleft lip and palate in the present outcome study?

In the present study, speech samples were assessed by means of auditory-perceptive analysis. This is considered to be the gold standard in the assessment of speech samples of patients with CLP [3,11]. The establishment of national databases for the research of cleft lip and palate (CRANE and SCANDCLEFT) has brought increased attention to the reliability and validity of auditory-perceptual analysis. In 2004 Lohmander and Olsen found a great heterogeneity between the variables [9], which led to unclear interpretations, especially given the small and heterogeneous samples, incomprehensible research designs and unclear implementations. Valid framework criteria are necessary, especially considering the individually varying auditory-perceptual capacities [14,29]. By adhering to methodological rigor, a study’s quality can be enhanced in terms of its validity [30]. Some authors also proved this aspect for auditory-perceptive analysis [9,10,11]. In the following section, the significance of these observations shall be assessed for the present study.

A look at the examined parameter ratings shows that there are obvious deviations in *nasal emission/nasal turbulence, hyponasality, voice function, acceptability, other oral misarticulations* and *weak articulation*. In all other categories a good to very good agreement was achieved. Several factors may have contributed to this:

First, it needs to be remarked that the underlying definitions and distinctions used in the translation of the URP-D [23] have not been aligned with those used at the international level [2]. All variables in consonant production errors apart from *oral misarticulations* and *weak articulation* showed full agreement. The reasons for disagreement seem, therefore, to be connected with deviant definitions. The differences in the ratings of *acceptability* may also be influenced by this. In LKGSF-Komplex, which is the theoretical basis for the German URP-D protocol, *intelligibility* and *acceptability* are not distinctly differentiated. The protocol itself gives a short description of the categories (see Table S2), but here, even the international URP version allows for a certain degree of individual interpretation.

The evaluation of *voice function* seems to be difficult, as can be seen in many studies (Sell et al., 2009; Bruneel et al., 2020) [14,16]. Wong et al. demonstrated that better outcomes in the auditory-perceptual evaluation of voice function can be achieved following training [31]. In the present study, *voice function* was rated by R1 and R2 by the means of the RBH Index. The index is used for evaluating functional and organic voice disorders. To the best of our knowledge, the criterion validity of voice function in patients with CLP using the RBH index has not yet been tested. R3 rated voice function on the basis of the CAPS-A training course as “due to a structural and/or a functional problem at the level of the larynx” [2]. In functional or organic voice disorders, the aspects of resonance and subglottic pressure during phonation are interrelated phenomena. They are usually due to muscular hyper- or hypofunctions within the laryngeal muscle loop system or to organic alterations at the vocal fold level [32]. Yet, Howard & Lohmander and Henningsson et al. define voice disorders in patients with cleft malformations as hyperfunctional symptoms in contrast to resonance disorders [33]. However, there is no causal muscular dysfunction or organic alteration at the glottal level in patients with CLP; rather, the primary issue is structural malformation at the level of the velum or due to fistulas. This results in an increased air leakage, which in turn can lead to changes in air pressure at the glottis level. Further investigations should be pursued to enhance the distinct definition and improve the validity of the variables, particularly with regard to their practical applicability in everyday speech-therapy practice.

The deviations in *hyponasality* may have different reasons: the inclusion criteria for all participants was a total unilateral cleft. The structural deviation of the nasal septum towards the unaffected side of the nose was visually observable in the video for some of the participants. A (partial) obstruction of the nasal cavity thus appears plausible, which likely leads to the occurrence of hyponasal resonance. The absence of background information about upper respiratory tract infections of participants or the distorting effect of audio recordings (see further down) may have also played a part in the deviant evaluations of the raters.

The weak agreement in the category of *other oral misarticulations* may be explained by the lack of foreknowledge concerning the participant’s dental or occlusal status. Based solely on auditive perception, speech sounds in this category are difficult to distinguish from other types of misarticulations (e.g., abnormal backing place remains oral). In addition, because the examination took place prior to midface osteotomy, it is plausible that dental conditions may have influenced speech intelligibility. Palatal asymmetry can result in crossbite, which is a common issue with CLP [34]. Crossbite often leads to palatal or lateral realization of the sibilant speech sounds (e.g., /sch/, /s/). Such deviations would need to be documented within the category of *other misarticulations* according to the definition (see Table 1) related to dental anomalies. All participants were impaired with a unilateral cleft, 16 patients on the left and 4 on the right side. In their review Wadhwa et al. suggested that patients with a ULCP on the left side are more likely to have supernumerary teeth [35]. Most of the examined participants in our study belong to this group. It therefore could be that the side difference could affect speech function as well. To our best knowledge no investigation has been made to this day to evaluate possible consequences for speech function. Future studies should investigate whether intelligibility is influenced by the side of impairment in individuals with ULCP.

Auditory perception of hypernasality, hyponasality, nasal emission/nasal turbulence and consonant productions errors is highly dependent on the quality of the audio recording. The reason is that not only do phonetic speech sounds have to be differentiated and evaluated, but so do airflow turbulences and non-speech acoustic noises. These signals may lie outside the frequency range of microphones designed for speech transmission. Speech samples were collected via Zoom™ using the built-in microphones of the respective computers. Thus, the conditions for a valid audio recording could not be achieved. This represents a significant influencing factor. The onset of sentences or words might reveal audible airflow turbulence but leave unclear whether the source was poor microphone quality, clipping due to close microphone proximity, or actual nasal emission/nasal turbulence. The problem is well known [17,18,36]. The recording of a standardized speech sample should be therefore as follows: The collection of the speech sample should be conducted in a quiet room, filmed against a calm background, with the participant’s face and neck illuminated by natural light, and recorded in digital quality. The speech sample should be captured using a unidirectional, professional condenser microphone placed at a minimum distance of 20–30 cm in front of and to the side of the patient. The microphone should be placed at the participant’s mouth level. Subsequent analysis should be conducted via room loudspeakers [2,12,14,36]. Current research tries to improve quality by further enhancement of the recordings [37]. Although in the present study consistent conditions were maintained on the side of the examiner, the aforementioned conditions could not be ensured during recording with Zoom™ in the participants’ home settings. Consequently, the variables of hypernasality, hyponasality, nasal emission/nasal turbulence as well as consonant production errors are subject to potential bias.

The content and structural design of the speech sample form the basis of the auditory-perceptual analysis and must therefore meet the highest quality standards [14]. The construction of the speech sample should ensure that individual target speech sounds can be distinctively perceived and structurally analyzed. Henningsson et al. advise the following conditions: in addition to 25–30 single words, testing should also include 15–20 sentences to assess hypernasality, nasal emission/nasal turbulence and articulation errors and 3–5 sentences to evaluate hyponasality, further spontaneous speech, automated speech and syllable repetition [2]. The main focus will be on high-pressure consonants (HPC) and vocals, because those are the primary sounds which become impaired in the speech of persons with CLP due to velopharyngeal insufficiency [38]. They occur in nearly all languages [2]. They thus represent typical speech characteristics of cleft lip and palate speech disorder and form the basis for global comparability of studies. Strong reliability could be demonstrated for the HPC [39]. Those vulnerable speech sounds should be embedded in a linguistic context, but must under no circumstances be coarticulatorily distorted by ambient speech sounds [40]. This concerns not only the sounds immediately surrounding the target phonemes but also the linguistic context in which the target phonemes are represented. Klintö et al. recommend embedding the target phonemes within words and sentences to achieve good reliability [41]. In the present study, the speech sample consisted of single words, sentences and spontaneous speech. The single words and sentences represented in the German version of GOS.SP.ASS have not yet been evaluated, and the development of the sentences has not been clearly documented. In fact, the words and sentences do not fully comply with the criteria set forth by Henningsson et al.; for example, the phonemes are not tested in all positions which occur in the German language. In some places an infection of the target phonemes cannot be excluded. For example, in the sentence “Kim bestellt Kuchen und kalten Kakao”, the phoneme /k/ is not tested in the final position although it occurs there regularly in the German phoneme system. The initial and medial position of the /k/ is possibly infected by other HPC in the sentence, such as b/, /s/, /t/ and /ch/. As a result, the target phoneme /k/ cannot be clearly and systematically perceived. This impairs both the speakers’ pronunciation of the target sound as well as the listener’s ability to analyze it.

In the present study, no automated speech or syllable repetition were tested in addition to the words and sentences. Since the validated CAPS-A protocol does not test all speech domains either [12], this might not have a profound influence on the results of global research. Further investigations should verify this.

In our study, words and sentences were supplemented by a guided interview aimed at eliciting spontaneous speech. The objective is to obtain a most authentic impression of everyday speech function. Although the questioning became standardized as such, the questions were not fully open-ended. The examination of spontaneous speech should always be conducted using open-ended questions [38] and elicit speech which as closely as much reflects everyday communication. For example, the question “What do you do for a living?” can be answered briefly with a few words, whereas a question such as “How would you promote your profession to young people?” would have been more effective in gaining naturally spoken language. Due to the brief responses given by some participants during the spontaneous speech assessment, everyday speech function was not always convincingly represented.

Furthermore, the construction of the speech sample should follow a certain testing sequence [2,11,12]. The first item to be listened to should be the audio recording of spontaneous speech, then the other speech domains and finally sentences. This ensures that the examiners cannot become accustomed to or anticipate the participant’s speech. The present study did not follow this sequence. The listeners first heard single words, then sentences and finally spontaneous speech. Consequently, auditory perception during spontaneous speech analysis may have been influenced by prior familiarization. Additionally, the URP-D protocol does not always follow the Henningsson protocol in the different domains. So, in contrast to URP-D, spontaneous speech in the original URP is only analyzed in voice disorder, understandability and acceptability but not in hypernasality, hyponasality, nasal emission/nasal turbulence or consonant production errors (see Tables S2 and S3). Overall, the applied design for auditory perception leads to significant deviations from internationally validated assessment practices.

Due to the complexity of the analysis, it is essential that the speech sample undergoes a systematic and structured evaluation [2,12,40]. Every single measuring point should be gained from a certain part of the speech sample. For example, in the sentences, only the target sound is rated, not the other possibly parallel phenomena which may occur. John et al. [12] conducted an audio as well as a video recording. In the audio format, the evaluation of the automated speech included assessments of voice quality, hypernasality, hyponasality, and nasal emission/nasal turbulence. For the sentences, which were also assessed auditorily, the target phonemes in each sentence were transcribed according to the International Phonetic Alphabet (IPA). Then, the automated sentences were presented again, this time in video. Here, scoring should be re-assessed a second time. In Great Britain examiners from different cleft centers are exchanged to conduct audits jointly. In this case the speech samples are first evaluated individually and blinded by three trained SLTs and later discussed in a consensus process. This procedure attained more validity in the auditory-perceptive analysis [42] and is currently said to be the gold standard for gathering valid data by means of auditory-perceptive analysis. In Germany a structured listening protocol has not yet been established. This inevitably leads to the development of individualized evaluation practices.

There is now broad consensus about the necessity of training to ensure a reliable analysis of speech samples in patients with cleft malformations. The purpose of the training in auditory-perceptual analysis is to enable center-independent evaluations and to promote national standardization. Recently, research has focused on developing models for an international training protocol for speech samples of patients with CLP; the objective is to gain high-quality cross-country studies [43]. Although it seems obvious that examination quality is higher in experienced than in unexperienced listeners [44,45], regular updated training is advised, even for experienced listeners, to develop unified and standardized categories [10,46]. This includes the regular training of phonetic transcription of speech samples, even with experienced listeners [10,14,18]. It was demonstrated that systematic training significantly improves interrater reliability [14,15,47]. To date, no systematic training exists in Germany, nor was there joint informal training among the three listeners before the present study. Without joint training, it was not possible to establish shared rating categories. As a result—despite high agreement in many areas—the two examiner groups produced differing results.

The intrarater reliability showed a very good correlation in the individual listeners at both scale levels. R1 and R2 belong to the same cleft center. Together they reached an intrarater reliability of 1.000 in ordinal scales. R3 works in a private practice from a different state and shows corresponding deviations, especially in rating nominal scales (hyponasality and voice disorder). High intrarater reliability values have been well documented [15,16]. It has been discussed that individual rating criteria may lack general validity due to prior experiences [15] or center-specific evaluation habits [14] which can introduce bias. This can lead to good intrarater reliability while interrater reliability shows only moderate results. The present study reveals a similar pattern.

The scale levels underlying the commonly used assessments (GOS.SP.ASS, CAPS-A, and consequently also the URP) alternate between ordinal scales (*hypernasality, nasal emission/nasal turbulence, intelligibility, acceptability*) and nominal scales (*hyponasality, voice and consonant production errors*). This results in different statistical calculation methods. An overall score across all variables is not possible to calculate. Various authors have employed different strategies to account for these discrepancies. The ICC was proposed for calculating all parameters [16]. However, the ICC is only applicable with ordinal scale levels [25]. Other authors calculated using kappa [12] or with ICC, kappa and percentual agreement [14]. Authors have discussed that the interpretability of ICC and kappa becomes limited when applied to small sample sizes or a low number of examiners. Viera & Garet point out that the calculation of a small group can confound the results if examiners use kappa [48]; this was verified by other authors [15,16]. The statistical problem deriving from the different scale levels and the low number of examiners can be confirmed through this study as well. The calculation of the interrater reliability in single measurements with ICC or Fleiss’ kappa [25] (see Table 2 and Table 3) may be technically feasible, but its interpretation value remains limited. The statistical proximity to the level of random chance appears to be so high that meaningful correlations seem to be difficult. The differences between the participants and the severity of their symptoms were too small to be effectively captured by the statistical methods applied, due to the character of the outcome study. Given an insufficient prevalence of data [14] or a too minor diversity of variables [15], the authors proposed to apply, instead of ICC or kappa, percentual agreement for the analysis of data. For the present study, percentual agreement appears to give the most reasonable representation in relation to underlying raw data. Altogether 88.3% agreement was achieved. However, this calculation does not take into account the probability of agreement by chance. Castick et al. [49] studied the interrater and intrarater reliability by means of visual analog scaling (VAS). On the basis of the GOS.SP.ASS sentences, 30 audio recordings of patients with CLP were analyzed by five listeners focusing on *hypernasality, hyponasality, nasal emission/nasal turbulence, intelligibility* and *acceptability*. A good interrater reliability was achieved for ordinal scales [49]. Nevertheless, hyponasality showed an R^2^ of 0.504 in the linear model and 0.525 in the curvilinear model, representing the weakest results in the study. For this purpose, the scale level for hyponasality in the comparison group was converted from a nominal to an ordinal scale.

In summary, the present study highlights various factors influencing the calculated interrater reliability in the assessment of speech samples by means of the auditory-perceptual analysis. Reasons may be the definitions and distinctions among the variables, methodical rigor in construction of the speech sample, performance, constraints and analysis. The statistical options to indicate reliability are also limited in interpretation due to the scale levels and due to the small number of examiners as well as participants. Based on the issues discussed and in comparison with previous studies, the levels of agreement in this study are nevertheless good. Most importantly, they reflect the success of the multidisciplinary treatment outcome: the patients are highly intelligible and their speech function is well accepted.

Given the complexity of auditory-perceptive analysis and its varying influences on reliability, it seems reasonable to consider the development of neural networks in the context of artificial intelligence [50,51]. Even though indications of good reliability have been found, it should be noted that the quality of an artificial intelligence system depends on the input of valid data. Based on a standardized and validated version of the GOS.SP.ASS for German-speaking countries, it is conceivable that artificial intelligence could perform analyses for cross-center or global studies. However, hands-on therapeutic work remains indispensable in diagnosis and treatment, tailored to each patient and grounded in a valid speech sample and its evaluation.

### Limitations

Nevertheless, some general limitations need to be mentioned. Although all participants were treated with the same methods in a similar timeline and under comparable conditions at one German university hospital, there remain open questions. There is no insight in the manner and influence of the interdisciplinary work, which might have influenced the outcome. Some of the participants had previous speech therapy—others did not. Since the methods in speech therapy vary according to the needs of the patients, there might have been different treatments along the way. Documenting the different treatments and methods as benchmarks of the different partaking disciplines could lead to new aspects in future research concerning treatment outcome. In this study all patients were operated on with the Kriens protocol. We have no insight into whether a different surgical method would have had the same result. Cross-center studies could possibly enhance knowledge. All in all, sensible working together seems to be an important perspective in this matter.

## 5. Conclusions

To enhance the validity of future outcome studies within Germany, it is of critical importance to establish the highest possible reliability in auditory-perceptual analysis of speech samples in cases of CLP. Speech samples should be designed on the basis of GOS.SP.ASS sentences and of CAPS-A and be supported by robust criterion validity. The construction and performance of the speech sample should be based on internationally accepted definitions and follow international standards regarding content, sequence, implementation, and structured analysis in order to ensure alignment with and comparability to international research. Statistical data analysis should be based on sufficiently large sample sizes and numbers of raters. Continuous training in auditory-perceptual analysis should ensure and standardize the quality of the examiners’ rating abilities.

The speech outcomes of the present study are altogether very positive. However, these results can currently not be compared with other national or international studies, as no validated assessment criteria for auditory-perceptual analysis in cleft lip and palate speech disorders have yet been established in the German-speaking context.

## Figures and Tables

**Table 1:** Definition of the speech parameters according to Henningsson et al. (2008) [2].

Speech Parameters	Definitions	
**Hypernasality**	“… excessive nasal resonance heard on vowels and sometimes on voiced consonants” (p. 6)	
**Hyponasality**	“… decreased or insufficient nasal resonance heard on consonants and vowels” (p. 7)	
**Nasal** **Emission**	“… the audible escape of air through the nasal passage that accompanies, or is coproduced with, high-pressure consonants.” (p. 7)	
**Nasal** **Turbulence**	“… the audible escape of air into or through the nasal passage accompanying or coproduced with high-pressure consonants that generates a turbulent “snorting”“ (p. 7)	
**Voice** **disorder**	“… a deviation in voice characteristics due to structural and/or a functional problem at the level of the larynx” (p. 7)	
**Articulation** **errors**	abnormal backing to post-uvular	“pharyngeal and glottal production of a target oral pressure consonant” (p. 7)
	abnormal backing place remains oral	Replacement of the pressure target consonants backed but remaining oral (middorsum-palatal, velar, uvular) (p. 8)
	nasal fricative	Replacement of a target oral sound through the nasal airway. With or without turbulence (p. 8)
	nasal consonant for oral pressure consonant	Replacement of a target plosive consonant by a homorganic nasal consonant (p. 8)
	nasalized voiced pressure consonants	Can occur with moderate or severe hypernasality: nasalization of voiced plosives (p. 8)
	weak oral pressure consonants	General loss of power in the production of high-pressure consonants (p. 8)
	other misarticulations	Traditionally identified with dentofacial and oral structural deviations, e.g., lateralization, palatalization (p. 8)
	developmental articulation errors	Commonly observed in children without CLP, representing a delayed speech development (p. 8)

**Table 2:** Interpretation of kappa/weighted kappa, the intraclass coefficient (ICC) [28] and observed agreement.

Kappa/Weighted Kappa or ICC	Observed Agreement (%)	Clinical/Practical Relevance
<0.40	<70%	poor
0.40–0.59	70–79%	moderate
0.60–0.74	80–89%	good
0.75–1.00	90–100%	very good

**Table 3:** URP-D-assessed parameters and results presented as raw scores, with intraclass correlation coefficient (ICC) and percentage agreement (% A) relative to T2.

	n = 20	T1			T2				
		R1	R2	R3	R1	R2	R3	ICC	% A
**1**	**HN single words**							**0.31**	**100**
	within normal limits	19	19	19	19	19	19		
	mild	1	1	1	1	1	1		
	moderate	0	0	0	0	0	0		
	severe	0	0	0	0	0	0		
**2**	**HN sentences**							**0.54**	**95**
	within normal limits	16	16	16	16	16	16		
	mild	3	3	4	3	3	4		
	moderate	1	1	0	1	1	0		
	severe	0	0	0	0	0	0		
**3**	**HN spontaneous speech**							**0.54**	**95**
	within normal limits	16	16	15	16	16	16		
	mild	3	3	5	3	3	4		
	moderate	1	1	0	1	1	0		
	severe	0	0	0	0	0	0		
**4**	**NE/NT single words**							**0.36**	**80**
	within normal limits/none	9	9	13	9	9	13		
	present: intermittent/variable	11	11	7	11	11	7		
	present: frequent/pervasive	0	0	0	0	0	0		
**5**	**NE/NT sentences**							**0.53**	**85**
	within normal limits/none	8	8	12	8	8	11		
	present: intermittent/variable	11	11	7	11	11	8		
	present: frequent/pervasive	1	1	1	1	1	1		
**6**	**NE/NT Spontaneous speech**							**0.5**	**65**
	within normal limits/none	8	8	13	8	8	15		
	present: intermittent/variable	11	11	7	11	11	5		
	present: frequent/pervasive	1	1	0	1	1	0		
**7**	**Intellegibility**							**0.52**	**100**
	within normal limits	17	17	17	17	17	17		
	mild	3	3	3	3	3	3		
	moderate	0	0	0	0	0	0		
	severe	0	0	0	0	0	0		
**8**	**Speech acceptability**							**0.2**	**85**
	within normal limits	17	17	14	17	17	14		
	mild	3	3	5	3	3	5		
	moderate	0	0	1	0	0	1		
	severe	0	0	0	0	0	0		

Values of the URP-D parameters/variables assessed by raters for hypernasality (HN), nasal emission (NE) and nasal turbulence (NT), speech intelligibility, and acceptability of verbal utterances at two assessment time points (T1 and T2).

**Table 4:** Values of the URP-D parameters presented as raw scores, with Fleiss’ kappa and percentage agreement (% A) relative to T2.

	n = 20	T1			T2				
		R1	R2	R3	R1	R2	R3	Fleiss’ Kappa k	% A
**9**	**HY sentences**							**0.37**	**70**
	within normal limits/none	17	16	10	17	17	11		
	present	3	4	10	3	3	9		
**10**	**HY spontaneous speech**							**0.35**	**80**
	within normal limits/none	17	16	13	17	16	13		
	present	3	4	7	3	4	7		
**11**	**Voice disorder**							**0.07**	**90**
	within normal limits/none	19	19	16	19	19	17		
	present	1	1	4	1	1	3		

Values of the URP-D parameters/variables assessed by raters for hyponasality (HY) and voice disorder at two assessment time points (T1 and T2).

**Table 5:** Values of the URP-D parameters and results presented as raw scores, with percentage agreement (%A) relative to T2.

Consonant Production Errors	T1			T2			
n = 20	R1	R2	R3	R1	R2	R3	%A
**SINGLE WORDS**							
**abnormal backing: oral to post-uvular (pharyngeal)**							**100**
within normal limits/none	0	0	0	0	0	0	
present	20	20	20	20	20	20	
**abnormal backing: oral to post-uvular (glottal)**							**100**
within normal limits/none	0	0	0	0	0	0	
present	20	20	20	20	20	20	
**abnormal backing, remaining oral to middorsum-palatal**							**80**
within normal limits/none	4	4	0	4	4	0	
present	16	16	20	16	16	20	
**abnormal backing, remaining oral to velar**							**90**
within normal limits/none	2	2	0	2	2	0	
present	18	18	20	18	18	20	
**abnormal backing, remaining oral to uvular**							**100**
within normal limits/none	0	0	0	0	0	0	
present	20	20	20	20	20	20	
**nasal fricative, phonem specific**							**95**
within normal limits/none	0	0	1	0	0	1	
present	20	20	19	20	20	19	
**nasal fricative, not phonem specific**							**100**
within normal limits/none	0	0	0	0	0	0	
present	20	20	20	20	20	20	
**nasal consonant for oral pressure consonant**							**100**
within normal limits/none	0	0	0	0	0	0	
present	20	20	20	20	20	20	
**nasalized voiced pressure consonants**							**95**
within normal limits/none	0	0	1	0	0	1	
present	20	20	19	20	20	19	
**weak articulation**							**60**
within normal limits/none	8	8	0	8	8	0	
present	12	12	20	12	12	20	
**other oral misarticulations**							**30**
within normal limits/none	16	17	3	16	17	3	
present	4	3	17	4	3	17	
**developmental articulation error**							**100**
within normal limits/none	0	0	0	0	0	0	
present	20	20	20	20	20	20	
**SENTENCES**							
**abnormal backing: oral to post-uvular (pharyngeal)**							**100**
within normal limits/none	0	0	0	0	0	0	
present	20	20	20	20	20	20	
**abnormal backing: oral to post-uvular (glottal)**							**100**
within normal limits/none	0	0	0	0	0	0	
present	20	20	20	20	20	20	
**abnormal backing, remaining oral to middorsum-palatal**							**100**
within normal limits/none	0	0	0	0	0	0	
present	20	20	20	20	20	20	
**abnormal backing, remaining oral to velar**							**100**
within normal limits/none	0	0	0	0	0	0	
present	20	20	20	20	20	20	
**abnormal backing, remaining oral to uvular**							**100**
within normal limits/none	0	0	0	0	0	0	
present	20	20	20	20	20	20	
**nasal fricative, phonem specific**							**95**
within normal limits/none	0	0	1	0	0	1	
present	20	20	19	20	20	19	
**nasal fricative, not phonem specific**							**100**
within normal limits/none	0	0	0	0	0	0	
present	20	20	20	20	20	20	
**nasal consonant for oral pressure consonant**							**100**
within normal limits/none	0	0	0	0	0	0	
present	20	20	20	20	20	20	
**nasalized voiced pressure consonants**							**90**
within normal limits/none	2	2	0	2	2	0	
present	18	18	20	18	18	20	
**weak articulation**							**65**
within normal limits/none	7	7	0	7	7	0	
present	13	13	20	13	13	20	
**other oral misarticulations**							**15**
within normal limits/none	16	17	0	16	17	0	
present	4	3	20	4	3	20	
**developmental articulation error**							**100**
within normal limits/none	0	0	0	0	0	0	
present	20	20	20	20	20	20	
**SPONTANEOUS SPEECH**							
**abnormal backing: oral to post-uvular (pharyngeal)**							**100**
within normal limits/none	0	0	0	0	0	0	
present	20	20	20	20	20	20	
**abnormal backing: oral to post-uvular (glottal)**							**100**
within normal limits/none	0	0	0	0	0	0	
present	20	20	20	20	20	20	
**abnormal backing, remaining oral to middorsum-palatal**							**100**
within normal limits/none	0	0	0	0	0	0	
present	20	20	20	20	20	20	
**abnormal backing, remaining oral to velar**							**100**
within normal limits/none	0	0	0	0	0	0	
present	20	20	20	20	20	20	
**abnormal backing, remaining oral to uvular**							**100**
within normal limits/none	0	0	0	0	0	0	
present	20	20	20	20	20	20	
**nasal fricative, phonem specific**							**100**
within normal limits/none	0	0	0	0	0	0	
present	20	20	20	20	20	20	
**nasal fricative, not phonem specific**							**100**
within normal limits/none	0	0	0	0	0	0	
present	20	20	20	20	20	20	
**nasal consonant for oral pressure consonant**							**100**
within normal limits/none	0	0	0	0	0	0	
present	20	20	20	20	20	20	
**nasalized voiced pressure consonants**							**90**
within normal limits/none	2	2	0	2	2	0	
present	18	18	20	18	18	20	
**weak articulation**							**70**
within normal limits/none	6	6	0	6	6	0	
present	14	14	20	14	14	20	
**other oral misarticulations**							**30**
within normal limits/none	16	17	3	16	17	3	
present	4	3	17	4	3	17	
**developmental articulation error**							**100**
within normal limits/none	0	0	0	0	0	0	
present	20	20	20	20	20	20	

Values of the URP-D parameters assessed by raters for articulation at two assessment time points T1 and T2.

**Table 6:** Intrarater reliability individual measures (ICC and weighted kappa).

Intrarater Reliability	R1	R2	R3
Ordinal scales ICC	1.000	1.000	0.995
Nominal scales weighted kappa	1.000	1.000	0.76

## Data Availability

The data presented in this study are available on request from the corresponding author.

## Supplementary Materials

The following supporting information can be downloaded at: https://www.mdpi.com/article/10.3390/jcm15020588/s1, Table S1: Test protocol (modified according to Great Ormond Street Speech Assessment, 98-Germany Version, GOS.SP.ASS 98-D; Table S2: Universal parameter ratings for reporting speech outcomes in cleft palate, URP-D according to Neumann [23]; Table S3: Universal parameter ratings for reporting speech outcomes in cleft palate, URP-D according to Henningsson et al. [2].

## Abbreviations

The following abbreviations are used in this manuscript:

CAPS	Cleft audit protocol for speech
CAPS-A	Cleft audit protocol for speech–augmented
CAPS-A VPC SUM	Cleft audit protocol for speech–augmented velopharyngeal composite summary Score
CLP	Cleft lip palate
CLISPI	Cleft palate international speech issues
CTC	Cleft type characteristics
GOS.SP.ASS	Great Ormond speech assessment
HN	Hypernasality
HPC	High-pressure consonants
HY	Hyponasality
ICC	Interclass coefficient
IPA	International phonetic alphabet
NE	Nasal emission
NT	Nasal turbulence
R	Rater
SLT	Speech and language therapist
URP	Universal parameters for reporting speech outcomes (in individuals with cleft palate)
URP-D	Universal parameters for reporting speech outcomes (in individuals with cleft palate)—German Version
