# Supplementary material for: Reliability of Auditory-Perceptual Analysis in the Study of Speech Function in Patients with Unilateral Cleft and Palate
Source: J Clin Med. 2026 Jan 12;15(2):588. doi: 10.3390/jcm15020588 (PMC12842387; doi:10.3390/jcm15020588)
Supplement: Supplementary file 1 [file jcm-15-00588-s001.zip › jcm-4050024-supplementary.pdf]

Supplement S1

Table S1: Test protocol (modified according to Great Ormond Street Speech Assessment 98-German version, GOS.SP.ASS 98-D)

1. single words

Mühle
Daumen
Puppe
Dieb
Baby
voll
Affe
Huf
Wolle
Löwe
Sonne
löschen
Lolly
Gabel
Tatze
Auto
Hut
Dach
Haus
Zaun
Herz
Schuh
Busch
Küche
Teich
Ring
Kuh
Hocker
Rock
Eule
Elefant

2. Sentences:

/m/:	*Mama möchte Mehl mahlen.*	*Nenne meine Mama Mimi.*
/p/:	Papa pustet die Pusteblume ab.	
/b/:	Bobby badet das Baby.	
/f/:	Vier Affen fallen vom Schiff.	
/v/:	Uwe will den Wagen waschen.	
/n/:	*Nina nimmt eine neue Ananas.*	*Neun Nonnen nennen nie einen Namen.*
/l/:	Lisa will mal einen Lolly.	
/t/:	Tim tut die Tafel in die Tüte.	
/d/:	Didi redet mit der Dame.	

/s/:	Rasmus soll das Glas nicht fallen lassen.
/z/:	Susi sieht sieben Hasen im Gras.
/r/:	Rolf schneidet Resi ihre roten Haare.
/ʃ/:	Der schöne Glitzerfisch schenkt dir eine Schuppe.
/tʃ/:	Mit lautem Klatsch fällt der Tscheche in den Matsch.
/ʒ/:	James fährt den Jeep in die Garage.
/ç/:	Michel sieht ein Eichhörnchen.
/ŋ/:	Inge schaut die lange Schlange an.
/k/:	Kim bestellt Kuchen und kalten Kakao.
/g/:	Gabi legt Gemüse auf die Waage.
/χ/:	Achim ist noch wach.
/h/:	Hanna hat einen fabelhaften Hut auf.
s-Cluster:	Stefan spritzt Wasser aus dem Fenster.
ks-Cluster:	Die Hexe fängt den Fuchs.
ts-Cluster:	Die Katze hängt im Netz.

3. Spontaneous speech:

Was machen Sie beruflich?

Haben Sie Freizeitaktivitäten?

Was sehen Sie am liebsten im Fernsehen/ Kino/ beim Streamen?

Worüber haben Sie sich zuletzt besonders gefreut oder geärgert?

Wer wohnt noch in Ihrem Haushalt?

Supplement S2

Table S2: Universal parameter ratings for reporting speech outcomes in cleft palate, URP-D according to Neumann, 2011

**Hypernasalität_Einzelwörter**		**Hypernasalität_Sätze_Spontansprache**	
0	unauffällig/im normalen Limit	0	unauffällig/im normalen Limit
1	leicht	1	leicht
2	moderat	2	moderat
3	stark	3	stark
-	fehlende Daten	-	fehlende Daten
**Hyponasalität_Sätze_Spontansprache**		**Stimmstörung_Komplette Sprachprobe**	
0	keine/im normalen Limit	0	keine/im normalen Limit
1	präsent	1	präsent
-	fehlende Daten	-	fehlende Daten
**Hörbarer nasaler Durchschlag und/oder Nasale Turbulenz_Einzelwörter**		**Hörbarer Nasaler Durchschlag und/oder Nasale Turbulenz_Sätze_Spontansprache**	
0	kein(e)/im normalen Limit	0	kein(e)/im normalen Limit
	präsent		präsent
1	variabel oder sporadisch/zeitweilig	1	variabel oder sporadisch/zeitweilig
2	konstant oder häufig	2	konstant oder häufig
-	fehlende Daten	-	fehlende Daten
**Artikulationsfehler_Einzelwörter**		**Artikulationsfehler_Sätze_Spontansprache**	
0	keine/im normalen Limit	0	keine/im normalen Limit
1	präsent	1	präsent

	artikulatorische Rückverlagerung an post-uvulare Stelle		artikulatorische Rückverlagerung an post-uvulare Stelle
	nach pharyngeal		nach pharyngeal
	nach glottal		nach glottal
	artikulatorische Rückverlagerung jedoch oral verbleibend		artikulatorische Rückverlagerung jedoch oral verbleibend
	nach middorsum-palatal		nach middorsum-palatal
	nach velar		nach velar
	nach uvular		nach uvular
	nasaler Frikativ		nasaler Frikativ
	phonem-spezifisch		phonem-spezifisch
	nicht phonem-spezifisch		nicht phonem-spezifisch
	ersetzung eines Fortis-Konsonanten durch einen Nasal		ersetzung eines Fortis-Konsonanten durch einen Nasal
	abgeschwächte Artikulation		abgeschwächte artikulation
	nasalierte stimmhafte Fortis-Konsonanten		nasalierte stimmhafte Fortis-Konsonanten
	andere orale Fehlartikulationen		andere orale Fehlartikulationen
	Ausspracheentwicklungsstörung		Ausspracheentwicklungsstörung
-	fehlende Daten	-	fehlende Daten
**Verständlichkeit_Spontansprache**		**Akzeptanz der sprachlichen Äußerungen_Komplette Sprachprobe/Spontansprache**	
0	im normalen Limit: Sprechweise ist unauffällig und akzeptiert	0	im normalen Limit: Sprechweise ist unauffällig und akzeptiert
1	leicht eingeschränkt: Sprechweise weicht leicht von der sozial akzeptierten Norm ab	1	leicht eingeschränkt: Sprechweise weicht leicht von der sozial akzeptierten Norm ab
2	moderat eingeschränkt: Sprechweise weicht deutlich von der sozial akzeptierten Norm ab	2	moderat eingeschränkt: Sprechweise weicht deutlich von der sozial akzeptierten Norm ab
3	stark eingeschränkt: Sprechweise weicht stark von der sozial akzeptierten Norm ab	3	stark eingeschränkt: Sprechweise weicht stark von der sozial akzeptierten Norm ab
-	fehlende Daten	-	fehlende Daten

Supplement S3

Table S3: Universal parameter ratings for reporting speech outcomes in cleft palate according to Henningsson et al., 2008

**Hypernasality_Single Words**		**Hypernasality_Sentences**	
0	within normal limits	0	within normal limits
1	mild	1	mild
2	moderate	2	moderate
3	severe	3	severe
X	missing data	X	missing data
**Hyponasality_Sentences**		**Voice Disorder_Whole Speech Sample**	
0	within normal limits/none	0	within normal limits/none
1	present	1	present
X	missing data	X	missing data
**Nasal Emission/Nasal Turbulence_Single Words**		**Nasal Emission/Nasal Turbulence_Sentences**	
0	within normal limits/none	0	within normal limits/none
	present (√) the frequency		present (√) the frequency
1	intermittent or variable	1	intermittent or variable
2	frequent or pervasive	2	frequent or pervasive
X	missing data	X	missing data
**Consonant Production Errors_Single Words**		**Consonant Production Errors_Sentences**	
0	within normal limits/none	0	within normal limits/none
	present, all that apply:		present, all that apply]:
	abnormal backing of oral targets to post uvular place		abnormal backing of oral targets to post uvular place
	to pharyngeal		to pharyngeal
	to glottal		to glottal
	abnormal backing of oral targets but remains oral		abnormal backing of oral targets but remains oral
	to mid-dorsum-palatal		to mid-dorsum-palatal
1	to velar	1	to velar
	to uvular		to uvular
	nasal fricative		nasal fricative
	phonem specific		phonem specific
	not phonem specific		not phonem specific
	nasal consonant for oral pressure consonant		nasal consonant for oral pressure consonant
	nasalized voiced pressure consonant		nasalized voiced pressure consonant
	weak oral pressures		weak oral pressures

	other misarticulations		other misarticulations
	developmental articulation/phonological error		developmental articulation/phonological error
X	missing data	X	missing data
**Speech Understandability_Conversational Speech**		**Speech Acceptability_Whole Speech Sample**	
0	within normal limits: speech is always easy to understand	0	within normal limits: speech is normal
1	mild: speech is occasionally hard to understand	1	mild: speech deviates from normal to a mild degree
2	moderate: speech is often hard to understand	2	moderate: speech deviates from normal to moderate degree
3	severe: speech is hard to understand most of the time	3	severe: speech deviates from normal to severe degree
X	missing data	X	missing data
